# The international Hip Outcome Tool 12 questionnaire (iHOT-12): an Italian language cross-cultural adaptation and validation

**DOI:** 10.1186/s10195-024-00796-w

**Published:** 2024-11-27

**Authors:** Filippo Randelli, Alberto Fioruzzi, Alice Clemente, Alessandra Radaelli, Alessandra Menon, Danilo Di Via, Clemente Caria, Manuel Giovanni Mazzoleni, Claudio Buono, Paolo Di Benedetto, Michele Paciotti, Rocco Papalia, Gennaro Fiorentino, Marco Collarile, Manlio Panasci, Christian Carulli, Marco Rosolani, Federico Della Rocca, Simona Cerulli, Domenico Potestio, Michele Conati, Alberto Costantini, Gian Nicola Bisciotti, Alessandro Aprato, Loris Perticarini, Nicola Santori, Mattia Loppini, Giuseppe Solarino, Giovanni Vicenti, Vito Pavone, Alberto Di Martino, Fabrizio Rivera

**Affiliations:** 1 Reparto di Chirurgia dell’Anca Displasica (CAD), ASST Centro Specialistico Ortopedico Traumatologico Gaetano Pini-CTO, University of Milan, Milan, Italy; 2 Scuola di Specializzazione in Ortopedia e Traumatologia, Università degli Studi di Milano, Via Festa del Perdono 7, 20122 Milan, Italy; 3 Laboratory of Applied Biomechanics, Department of Biomedical Sciences for Health, Università Degli Studi di Milano, Via Mangiagalli 31, 20133 Milan, Italy; 4 Clinica Ortopedica e Traumatologica Policlinico G. Rodolico - San Marco-Università degli Studi di Catania, Catania, Italy; 5 Ortopedia e Traumatologia – Università Campus Biomedico, Rome, Italy; 6 Università di Bari “Aldo Moro” - AOU Policlinico Consorziale, Bari, Italy; 7 Clinica Ortopedica Dipartimento di Medicina Università degli Studi di Udine, Milan, Italy; 8 Ospedale Humanitas Gavazzeni Bergamo, Bergamo, Italy; 9 San Carlo di Nancy Hospital - Gvm Care and Research, Rome, Italy; 10 Villa Stuart Sport Clinic FIFA Medical Centre of Excellence, Rome, Italy; 11 Clinica Ortopedica, Università di Firenze SOD Ortopedia - Centro Traumatologico Ortopedico, AOU Careggi, Firenze, Italy; 12 Chirurgia Protesica e Mininvasiva Anca e Ginocchio Humanitas Research Hospital, Rozzano, MI Italy; 13 Fondazione Policlinico Universitario Agostino Gemelli IRCCS, Rome, Italy; 14 Arsbiomedica, Rome, Italy; 15 Divisione di Ortopedia 1 Ospedale di Suzzara MN, Casa di Cura San Francesco, Verona, Italy; 16 Concordia Hospital, Rome, Italy; 17 Kinemove Rehabilitation Centers, Pontremoli, La Spezia, Italy; 18 Università di Torino, Torino, Italy; 19 Fondazione Poliambulanza Istituti Ospedalieri - Brescia, Brescia, Italy; 20 European Hospital, Rome, Italy; 21 Department of Biomedical Sciences, Humanitas University, Via Rita Levi Montalcini 4, Pieve Emanuele, Milan, Italy; 22 IRCCS Humanitas Research Hospital, Via Manzoni 56, Rozzano, MI Italy; 23 Department of Traslational Biomedicine and Neuroscience (DiBraiN), University of Bari Aldo Moro, AOU Consorziale Policlinico, Bari, Italy; 24 Dipartimento Chirurgia Generale e Specialità Medico-Chirurgiche, PO Policlinico Gaspare Rodolico, Università degli Studi di Catania, Catania, Italy; 25 1st Orthopaedic Department, IRCCS-Istituto Ortopedico Rizzoli, Via Giulio Cesare Pupilli, 1, 40136 Bologna, Italy; 26 Department of Biomedical and Neuromotor Science-DIBINEM, University of Bologna, 40126 Bologna, Italy; 27 SC Ortopedia e Traumatologia, Ospedale SS Annunziata Savigliano, ASL CN1, Cuneo, CN Italy

**Keywords:** Hip, Proms, iHOT12, Score, Validation, Italian

## Abstract

**Background:**

Patient-reported outcome (PRO) measures are essential for evaluating disease-related quality of life. The International Hip Outcome Tool 12 (iHOT12) assesses various aspects of hip-related symptoms, function, sports participation, and social limitations. This study aimed to adapt and validate an Italian version of the iHOT12 according to COnsensus-based Standards for the selection of health Measurement INstruments (COSMIN) guidelines.

**Materials and methods:**

A multicenter observational cohort study was conducted to translate and validate the iHOT-12 into Italian (iHOT-12-ita) and assess its psychometric properties. Following international guidelines, we translated and culturally adapted the iHOT-12-ita. Patients with hip pain included in the study completed the iHOT-12-ita and mHHS-ita questionnaires at three timepoints: initial consultation (T0), 10–20 days later (T1), and at least 2 months after treatment (T2): hip injection, hip arthroscopy or total hip replacement. The evaluation of the iHOT-12-ita psychometric properties was conducted following the COSMIN checklist, assessing its reliability, responsiveness, interpretability, and acceptability.

**Results:**

Between May 2019 to September 2022, 104 patients were included, from two tertiary hip surgery referral center clinics, they completed both questionnaires and were available for data analysis. The psychometric evaluations revealed robust test–retest reliability on 27 patients at T1, with a Lin coefficient of 0.709 [95% confidence interval (CI) 0.553–0.888] and a high Pearson correlation coefficient of 0.745. At T2 after treatment, there was significant responsiveness (*p* < 0.001, *n* = 72), with excellent correlation observed between the iHOT-12-Ita and mHHS-Ita questionnaires (pwcorr = 0.7971). All predefined hypotheses were confirmed.

**Conclusions:**

The Italian iHOT-12-ita proved reliable, valid, and responsive in assessing hip disease-related quality of life. Its implementation in routine clinical practice and research can enhance patient care and research quality in hip preservation surgery for the Italian orthopedic community.

*Level of evidence*: II.

**Supplementary Information:**

The online version contains supplementary material available at 10.1186/s10195-024-00796-w.

## Introduction

Hip joint preservation surgery (HJPS) has witnessed significant global expansion over recent decades, evidenced by a surge in the number of surgeries performed and increased research activity in the field. Since HJPS primarily targets young adults in pre-arthritic conditions, an effective evaluation of surgery outcomes necessitates the assessment of patients’ health-related quality of life (HRQoL) using appropriate tools. Consequently, several patient-reported outcome measures (PROMs) [1, 2] have been developed specifically to evaluate pre-arthritic hip conditions, with a particular emphasis on assessing physical activity, psychological aspects, social functions, and overall well-being.

The international Hip Outcome Tool 33 (iHOT-33) [3] stands out as a reliable and validated patient-reported outcome (PRO) designed to address non-arthritic hip-related problems in the active young adult population. Its abbreviated version, the iHOT-12 [4], has been validated to enhance its applicability in routine clinical practice. Owing to its ease of use in clinical settings and its focus on pre-arthritic hip conditions, thus aligning well with the active lifestyles of young patients, the iHOT-12 is experiencing rapid adoption in the research domain of HJPS. This adoption is further underscored even by its inclusion in the UK’s Non-Arthroplasy Hip Registry (NAHR) [5], where it serves as a valuable tool for assessing this particular patient demographic undergoing HJPS.

To our knowledge, the iHOT-12 has already undergone translation and validation into Portuguese [6], Swedish [7], Dutch [8], German [9], Japanese [10], Turkish [11], and French [12]. The primary objectives of this study were to develop the Italian translation of the iHOT-12 and to evaluate its psychometric properties, including test–retest reliability and responsiveness, as well as its interpretability and acceptability.

## Methods

### Study design

The study was designed as a multicenter observational cohort with prospective data collection. Initially, the English iHOT-12 questionnaire was translated into Italian and adapted to the Italian setting. Subsequently, its psychometric properties were evaluated in a prospective manner, encompassing assessments of test–retest reliability and responsiveness. Additionally, interpretability and acceptability were assessed. Throughout the translation and validation process, adherence to COnsensus-based Standards for the selection of health Measurement INstruments (COSMIN) guidelines and checklist was ensured [13–15]. All data collected during the study were handled in accordance with Italian legislation concerning personal data protection.

### Development of the Italian version of the HOOS: translation and cross-cultural adaptation

The Italian version of the iHOT-12 (iHOT-12-ita) was developed and culturally adapted from the translation of the original iHOT-12 questionnaire in accordance with published international recommendations and Beaton’s guidelines [16, 17]. The translation process comprised six phases: preparation, forward translation, reconciliation, backward translation, review and harmonization, and proofreading.

#### Preparation

The principal investigator of the study (F.R.) conducted checks within the Italian orthopedic community to ascertain whether any other ongoing translations of the iHOT-12 into Italian were in progress.

#### Forward translation and reconciliation

Two Italian native translators with diverse professional backgrounds independently translated the questionnaire into Italian. Subsequently, a consensus meeting was arranged to facilitate a discussion between the two translators, enabling them to review any discrepancies and reach agreement on a single unified version.

#### Backward translation

An English native translator, with no specific background in the health field and blinded to the original iHOT-12 English version, back-translated the questionnaire into English. The translator meticulously examined the back-translated version to identify any inconsistencies or conceptual errors.

#### Review and harmonization

Here is an improved version of your text with enhanced clarity and precision:

A multidisciplinary panel, consisting of 16 members from the Società Italiana di Artroscopia, Ginocchio, Arto Superiore, Sport, Cartilagine e Tecnologie Ortopediche (SIAGASCOT) Hip Committee, all experts in hip joint preservation surgery (HJPS), including orthopedic surgeons with documented experience in questionnaire validation, conducted a thorough review of the final text to ensure consistency in four fundamental areas:

1. Semantic equivalence: the committee verified whether the translated words retained the same meaning as the original text.
2. Idiomatic equivalence: the committee assessed whether any colloquialisms or idiomatic expressions were appropriately translated to ensure comprehension without altering the intended meaning.
3. Practical equivalence: the committee evaluated whether the questionnaire items effectively captured everyday life experiences relevant to the target population.
4. Understandability and transcultural adaptation: the committee ensured that the questionnaire was easily understandable and culturally adapted for the Italian context.

Following meticulous scrutiny and discussion, the committee reached a consensus on the questionnaire’s final version, which underwent additional checks to confirm its understandability and transcultural adaptation.

#### Proof reading

Following the consensus on the questionnaire’s final version, a subgroup comprising ten patients experiencing hip pain underwent cognitive debriefing. This process aimed to test alternative wordings and assess the understandability, interpretation, and cultural relevance of the translation. After the completion of the cognitive debriefing phase and the incorporation of any necessary revisions, the final Italian version of the iHOT-12 (iHOT-12ita) was released (see Appendix A).

#### Final feed-back from scientific society

The final version of iHOT12 was then sent to Members of Hip and Groin Pain Committee of the major Sport Medicine Italian Scientific Society, SIAGASCOT (Italian society of arthroscopy, knee, upper limb, sport, cartilage, orthopedic technologies) for an expert feed-back obtaining a full approval and commitment to use iHOT12 in their clinical practice.

### Questionnaires

The iHOT12 was initially developed in 2012 by Griffith et al. [4] as a concise adaptation of the iHOT33 [3], aimed at facilitating routine clinical practice to measure the impact of hip disease in young, active patients and to assess the effectiveness of treatment. This self-administered questionnaire comprises 12 items categorized into four distinct patient-relevant dimensions: symptoms and functional limitations (items: 1, 2, 3, 4), sports and recreational activities (items: 5, 7, 11), job-related concerns (item: 6), and social, emotional, and lifestyle factors (items: 8, 9, 10, 12). Respondents describe their hip status and associated quality of life (QoL) by marking a point along a defined 100 mm horizontal line. Each question and its corresponding answer carry equal weight in determining the overall test result. Possible scores for each item range from 0 to 100, with the summation of each item’s scores resulting in a normalized overall score of 100 indicating excellent QoL and a score of 0 representing the poorest QoL. The Italian version developed, as described above, was utilized in the present study for validation purposes (see Appendix).

The Modified Harris Hip Score (mHHS) [18] stands as the most widely utilized patient-reported outcome measure (PROM) for hip diseases. Derived from the original Harris Hip Score [19], it was initially designed to assess patients with hip osteoarthritis both before and after treatment. The original HHS, designed in 1969, comprises ten items with a total score of 100 points, encompassing assessments of pain, functionality, absence of deformity, and range of motion (ROM). The mHHS is a modified version that retains eight of the original ten items, excluding the evaluation of ROM. The maximum achievable score for the mHHS is 91 points, which is then normalized to 100, indicating excellent quality of life (QoL). In 2015, Dettoni et al. validated an Italian version of the HHS, which was utilized in the present study [20].

### Patient enrollment and questionnaires administration

The study population comprised patients recruited from May 2019 to September 2022 from two tertiary hip surgery referral center clinics. A validation threshold of 100 patients undergoing operative treatment was set. The target population consisted of patients experiencing hip pain and candidates for operative treatments such as hip intra-articular injection, hip arthroscopy, or total hip replacement.

The inclusion criteria were: patient age between 14 and 60 years, active lifestyle, and Italian native speakers.

The exclusion criteria were: patients unable to comprehend the Italian questionnaire; patients who did not undergo the indexed procedure (injection, arthroscopy, or arthroplasty); patients suffering hip diseases including infections, trauma, tumors, and dysplasia; insufficient reading and comprehension capacity; associated disorders of the back or the contralateral lower extremity; and individuals with drug addiction, alcoholism, psychiatric disorders, or clinical conditions that may compromise the success of the questionnaire.

All patients provided informed consent to participate in the study.

Patients’ demographic data were collected during the initial clinical consultation, along with the administration of self-administered questionnaires. Both the Italian versions of iHOT-12-Ita and the previously validated mHHS-Ita were administered and collected at three endpoints:

1. During the first clinical consultation (T0).
2. At 10–20 days after the clinical consultation, via email contact (T1).
3. At least 2 months after the operative procedure, either during a clinical consultation or via email (T2).

Once the questionnaires were completed, the data were standardized to a range from 0 to 100 and stored in a shared database. A total of 111 patients admitted to the orthopedic hip units of the Hip Units of two Italian hospitals were enrolled in an observational prospective multicenter setting. Table 1 summarizes patient characteristics. Out of the 111 eligible patients for follow-up, 7 were lost at follow up (6,3%).

**Table 1:** Demographic characteristics

Patient population, *n* (%)	Value
Total	111 (100%)
Excluded	7 (6.3%)
Included	104 (93.7%)
Male	55 (52.9%)
Female	49 (47.1%)
Age, average (± SD), years	
Total	45,0 (± 12.0)
Male	43,2 (± 12.2)
Female	47,0 (± 11.5)
Age stratification, *n* (%)	
≤ 20 years of age	2 (1.9%)
20–30 years of age	18 (17.3%)
30–40 years of age	12 (11.5%)
40–50 years of age	27 (26.0%)
50–60 years of age	45 (43.3%)
Affected hip, *n* (%)	
Right	52 (50%)
Left	52 (50%)
Diagnosis, *n* (%)	
Non arthritic hip pain (C-sign)	40 (38.5%)
Hip osteoarthritis	64 (61.5%)
Treatment, *n* (%)	
Hip injection	25 (24.0%)
Hip arthroscopy	26 (25.0%)
Hip arthroplasty	53 (51.0%)

Of the 104 patients who completed the initial iHOT12-ita questionnaire during the first consultation (T0), 27 agreed to complete it again via email contact before treatment to evaluate test–retest reliability at T1 (10–20 days after the initial self-administration). The mean time between T0 and T1 was 14.7 days (range: 10–20 days).

Furthermore, to assess iHOT-12-ita responsiveness and interpretability, 74 patients who responded at T0 completed the self-administered iHOT-12-ita again after treatment at T2 (via email contact, at least 2 months after T1). To evaluate convergent validity, all patients were requested to complete the mHHS-ita at each endpoint time.

### Statistical analysis and assessment of psychometric properties

#### Statistics

The statistical analysis was conducted using R Statistical Software (Version 4.0.0; R Foundation for Statistical Computing) and Prism software (Version 6.0; GraphPad Software Inc). Continuous variables were presented as either mean and standard deviation or median along with the first and third quartiles, as deemed appropriate. Categorical variables were expressed in terms of case numbers or frequencies.

#### Test–retest reliability

Test–retest reliability, is defined as “the extent to which scores for patients who have not changed are the same for repeated measurement […] over time” [13]. In our study, it was evaluated using the concordance correlation coefficient by Lin (CCC) with a 95% confidence interval (CI). The CCC combines measures of both precision and accuracy to ascertain how closely the observed data align with the line of perfect concordance (i.e., the line at a 45-degree angle on a square scatterplot). Lin’s coefficient increases in value as the data’s reduced major axis approaches the line of perfect concordance (indicating accuracy) and as the data cluster tightly around its reduced major axis (indicating precision). The concordance correlation coefficient (CCC), denoted as rho_c, can be expressed as the product of r (a measure of precision) and C_b (a measure of accuracy). Concordance is deemed good if rho_c > 0.60 and excellent if rho_c > 0.80.

A Bland–Altman plot was employed to assess agreement and the presence or absence of systematic error between the two scores (day 0 and day 14) of the two tests.

Finally the concordance of iHOT-12-Ita test at T0 and T1, was calculated also through the Pearson correlation.

#### Responsiveness

Responsiveness, is defined as “the ability of an HR-PRO instrument to detect change over time in the construct to be measured” [13]. This property assesses the questionnaire’s ability to capture genuine changes, essentially measuring its sensitivity to change when actual changes occur. In our study, responsiveness was evaluated by comparing the mean change in both PROMs (iHOT-12-Ita and mHHS-Ita) at T0 and T2 (before and after the operative intervention) using a paired *t*-test analysis.

Furthermore, the correlation between the data was assessed using Pearson correlation analysis. The Pearson correlation coefficient (*r*) was considered moderately high when *r* > 0.5, high when *r* > 0.7, and very high when *r* > 0.9. This analysis helped determine the strength and direction of the relationship between the changes observed in the two PROMs.

### Assessment of interpretability and acceptability

#### Interpretability

According to the COSMIN criteria [13], interpretability is not considered a measurement property but is regarded as an important characteristic of a measurement instrument. It refers to the degree to which one can assign qualitative meaning, such as clinical or commonly understood connotations, to an instrument’s quantitative scores or change in scores.

In our study, interpretability was assessed by calculating floor and ceiling effects for each score of iHOT-12-ita and mHHS-ita at T0 and T2. Floor and ceiling effects occur when a high proportion of participants score at the lowest or highest possible levels, respectively. A priori values for floor and ceiling effects were defined as > 15% of participants responding with the lowest or highest possible score, respectively [9, 12, 21, 22]. These analyses helped evaluate the extent to which the instruments’ scores were able to capture the full range of possible responses and provided insights into their interpretability.

#### Acceptability

Acceptability of the iHOT-12-ita was investigated at T0 by measuring the average time needed to fill out the questionnaire, the proportion of missing answers, and the self-confidence of patients in compilation. A compilation time < 15 min was considered satisfactory [23]. Referring to the proportion of missing answers and patients needing support in compilation, *a* < 5% value was assumed to be acceptable.

## Results

From of the 111 eligible patients for follow-up, 7 were lost at follow up (6,3%). The analysis of results has been performed on a population of 104 patients.

### Assessment of psychometric properties

#### Test–retest reliability

The concordance of iHOT-12-Ita test at T 0 and T1, as derived through the Lin coefficient, was good (rho_c = 0.709, 95% CI 0.553–0.888) compared with the high mHHS-Ita concordance (rho_c = 0.881, 95% CI 0.795–0.966).

The concordance of iHOT-12-Ita test at T0 and T1, calculated through the Pearson correlation coefficient was high (*r* = 0.745) and comparable to the mHHS-ita concordance at the same endpoints (*r* = 0.882).

No systematic error was present, based on the Bland–Altman plot.

#### Responsiveness

Responsiveness was tested by comparison of the scores at T0 and T2 of 74 patients who answered the questionnaires at both endpoint time. The paired *t*-test analysis displayed a mean increase of 39 (*p* < 0.001, *n* = 72) from T0–T2 according to iHOT-12-Ita. The mean difference between mHHS-Ita at T0 and T2 was 32 points (*p* < 0.001 *n* = 72).

All results showed an excellent correlation between the iHOT-12-Ita and mHHS-Ita questionnaires (pwcorr = 0.7971, n = 104).

### Assessment of interpretability and acceptability

#### Interpretability

Data from 79 patients were assessed to measure interpretability. Floor effect was found at T0 (104 patients) assessment in 0,96% for iHOT-12-ita and 1,92% for mHHS-ita.

Ceiling effect was found at T2 assessment (72 patients after intervention) in 5,56% for iHOT-12-ita and 23,61% for mHHS-ita.

#### Acceptability

Acceptability was tested on the preoperative administration (T0) of the iHOT-12-ita (104 patients). On average, the time to fill out the questionnaire was 7 min. No missing data were registered for any item nor assistance have been required.

## Discussion

In the present study, the development and assessment of the Italian version of iHOT-12 were conducted, focusing on evaluating its psychometric properties. The translation into Italian and cross-cultural adaptation resulted in patients reporting satisfaction with reading and understanding the iHOT-12-ita items (see Appendix). The psychometric properties indicated that the presented Italian version of iHOT-12 is a valid and reliable tool for assessing outcomes in patients suffering from hip pain owing to various hip conditions.

Consistent with findings from other translated versions (ICC = 0.84–0.94) [6–12] and the original iHOT-12 (ICC = 0.89), the test–retest reliability, calculated through the Pearson correlation coefficient (*r* = 0.745), was high. This indicates not only that the iHOT-12-ita is reliable, similar to the mHHS-ita (*r* = 0.882), but also suggests its stability over time.

The concordance of the iHOT-12-Ita test at T0 and T1, as derived through the Lin coefficient, yielded inferior outcomes, likely owing to the small proportion of the sample size at T1, which represents the first main limitation of the present study.

Responsiveness is another important property for patient-reported outcome measures (PROMs), measuring whether the difference obtained by the questionnaires is directly related to a clinical change. Similar to other translations of iHOT-12 [6–12], the Italian version designed in the present study demonstrated good responsiveness by comparing the clinical status at the time of the first evaluation (T0) and the improved outcomes at T2 after the operative intervention (*p* < 0.001). The presented Italian translation of the iHOT-12 also exhibited excellent correlation with the responsiveness of mHHS-ita (pwcorr = 0.7971). In this study, both PROMs results demonstrated very high sensitivity in highlighting a clinical difference, comparable to the original iHOt-12 [4] and its other transcultural adaptations [6–12].

Another quality criterion for content validity is the absence of floor and ceiling effects. Consistent with previous translations [6–12] and the original iHOT-12 [4], no relevant floor effect was observed for the iHOT-12-ita, as only one patient scored a value of zero (0.96%) at T0. Similarly, relevant ceiling effects were minimal, with only four patients (5.56%) achieving the maximum score of 100 after operative treatment at T2.

The presented Italian version of iHOT-12 proved to be acceptable, being easily understandable for each item and capable of being self-administered in about 7 min.

However, data collection presented some difficulties. While collecting data from several hospitals could be viewed as a limitation of the study, it provided a valuable opportunity to test the iHOT-12-ita in a real-clinical-practice setting rather than an experimental one. For example, during the assessment of responsiveness and interpretability, a response bias was detected in one hospital unit, leading to higher values for postoperative measurements. This highlights the importance of conducting assessments in diverse clinical settings to capture variations in patient populations and contexts.

Another potential limitation may arise from the decision to include patients with hip pain who were candidates for various operative treatments, such as hyaluronic acid infiltration, hip arthroscopy, and total hip replacement. Unlike other studies that focused on a single pathology or procedure [7, 10, 12], the rationale behind this choice was to ensure and validate the use of this PROM in a wide cohort of Italian-speaking patients with hip pain. This approach aimed to test the reliability and sensitivity of the iHOT-12 not only in young populations but also in the diverse clinical practice settings. Furthermore, the authors’ definition of hip pain, based on the presence of the C-sign, may introduce bias in patient selection owing to the subjective nature of symptom interpretation. This reliance on a patient’s self-reported C-sign could lead to variability in diagnosis, potentially affecting the study’s consistency and accuracy. However, it is important to note that the presumed bias stemming from the inclusion of patients undergoing various operative interventions and pain origin is mitigated by the absence of relevant ceiling effects observed for the iHOT-12-ita, even after different operative interventions, in contrast to the mHHS. This suggests that the iHOT-12-ita may offer a more comprehensive and sensitive assessment tool across a range of hip conditions and treatments, thereby enhancing its utility in clinical practice and research settings.

A possible limitation could arise from the methodology of questionnaire administration, whether in outpatient settings or via email contact. Standardizing the questionnaire submission could help mitigate potential response bias.

Notwithstanding the primary limitation of this study is undoubtedly the challenge of data collection. By the end of recruitment, seven patients were lost to follow-up (6,3%) because they did not undergo the scheduled treatment. However, the most significant dropout effect was observed at the T1 endpoint, during the retest PROM submission 10–20 days after the initial clinical evaluation. The low number of patients who responded to the retest (25.96% of the overall study population) did not allow for a complete statistical analysis, potentially resulting in lower concordance reliability compared with literature.

Despite these limitations, this study represents the first attempt to adapt and validate the iHOT-12 in Italy, thereby contributing valuable insights to the field of HJPS.

## Conclusions

The English version of the iHOT-12 questionnaire was successfully translated into Italian and transculturally adapted following international guidelines. The Italian version, iHOT-12-ita (see Appendix), has proven to be a valid and reliable tool for assessing patient-reported outcomes and the effectiveness of clinical interventions in individuals with hip pain, regardless of the type of treatment received. Is has been proven that iHOT-12-ita is comparable to the original iHOT-12 and the others transcultural adaptations.

The successful translation and validation of the iHOT-12 into Italian offers clinicians and researchers in Italy a standardized instrument that is highly beneficial for both clinical practice and future research in the field of hip preservation surgery.

## Supplementary Information

Supplementary material 1.

## Data Availability

Not applicable.
