# Supplementary material for: The international Hip Outcome Tool 12 questionnaire (iHOT-12): an Italian language cross-cultural adaptation and validation
Source: J Orthop Traumatol. 2024 Nov 27;25:59. doi: 10.1186/s10195-024-00796-w (PMC11602895; doi:10.1186/s10195-024-00796-w)
Supplement: Supplementary file 1 — Supplementary material 1. [file 10195_2024_796_MOESM1_ESM.pdf]

APPENDICE

# iHOT[12]

**INTERNATIONAL**
**HIP OUTCOME TOOL - 12**
**VERSIONE ITALIANA**

**NOME E COGNOME**

**DATA**

**Quale anca considera il questionario?**
Se ti abbiamo chiesto di compilarlo per una specifica anca, seleziona quel lato di seguito. Altrimenti seleziona il lato che causa maggiormente problemi.

○ **Sinistra**
○ **Destra**

QUESTIONARIO SULLA QUALITÀ DELLA VITA PER GIOVANI ATTIVI CON PROBLEMI ALL'ANCA

## ISTRUZIONI

- Le domande sono state formulate di modo che tu possa indicare quanto è grave il tuo problema. Le domande sono relative a vari problemi che potresti riscontrare all'anca, come questi influenzano la tua vita, e lo stato emotivo.
- Si prega di indicare la gravità **contrassegnando** la riga sotto ciascuna domanda con una **barra**.
  - » Se metti un segno all'estrema **sinistra**, significa che ritieni di essere notevolmente compromesso. Per esempio:

    NOTEVOLMENTE COMPROMESSO /________________________________ NESSUN PROBLEMA

  - » Se metti il segno all'estrema **destra**, significa che non pensi di avere problemi all'anca. Per esempio:

    NOTEVOLMENTE COMPROMESSO ________________________________/ NESSUN PROBLEMA

  - » Se il segno è posizionato al **centro** della linea, ciò indica che hai una invalidità moderata, in altre parole, tra gli estremi di "notevolmente compromesso" e "nessun problema". È importante mettere il segno alle due estremità della linea se le descrizioni estreme riflettono accuratamente la tua situazione.

**SUGGERIMENTO**
Se non svolgi un'attività, immagina come si sentirebbe la tua anca se dovessi provarla

- Rispondi ad ogni domanda pensando allo stato di salute della tua anca, considerando il **mese appena trascorso**.

---

Q1 Complessivamente quanto dolore hai alla tua anca/inguine?

DOLORE ESTREMO ________________________________ NESSUN DOLORE

---

Q2 Quanto è difficile per te alzarti e sederti per terra/sul pavimento?

ESTREMAMENTE DIFFICILE ________________________________ NESSUNA DIFFICOLTA'

---

Q3 Quanto è difficile per te camminare per lunghe distanze?

ESTREMAMENTE DIFFICILE ________________________________ NESSUNA DIFFICOLTA'

Q4 Quanti problemi hai avvertendo scricchiolio, cigolio, scatto o altri rumori dell'anca?

SERI PROBLEMI ______________________ NESSUN PROBLEMA

Q5 Quanti problemi hai nello spingere, tirare, alzare o portare oggetti pesanti?

SERI PROBLEMI ______________________ NESSUN PROBLEMA

Q6 Quanto sei preoccupato/a nello scattare/cambiare direzione durante le attività sportive o ricreative?

ESTREMAMENTE PREOCCUPATO/A ______________________ PER NIENTE PREOCCUPATO/A

Q7 Quanto dolore provi all'anca *dopo* le attività?

DOLORE ESTREMO ______________________ NESSUN DOLORE

Q8 Quanto ti preoccupa la situazione della tua anca quando prendi in braccio un bambino o cammini con un bambino in braccio?

ESTREMAMENTE PREOCCUPATO/A ______________________ PER NIENTE PREOCCUPATO/A

Q9 Quanti problemi hai con l'attività sessuale a causa della tua anca?

☐ Non è rilevante per me

SERI PROBLEMI ______________________ NESSUN PROBLEMA

Q10 Quanto spesso sei consapevole della disabilità della tua anca?

COSTANTEMENTE CONSAPEVOLE ______________________ NON LO NOTO AFFATTO

Q11 Quanto sei preoccupato/a riguardo la capacità di mantenere il tuo livello di forma fisica desiderato?

ESTREMAMENTE PREOCCUPATO/A ______________________ PER NIENTE PREOCCUPATO/A

Q12 Quanto ti distrae il tuo problema all'anca durante le attività giornaliere?

DISTRAE MOLTISSIMO ______________________ NON DISTRAE AFFATTO

***Per gli operatori:*** *ogni riga è lunga 10 cm. A seconda di dove viene apposto il segno bisogna misurare la lunghezza del segno rispetto al punto di origine e convertire la misura in un punteggio da 0 a 100.*

*Es.: il segno viene apposto a 7,7 cm dall'inizio della riga. Il punteggio sara' quindi di 77 punti.*

*Es.: il segno viene apposto a 4,3 cm dall'inizio della riga. Il punteggio sara' quindi di 43 punti.*

*Il* ***punteggio finale*** *dell'i-HOT12 si ottiene sommando i punteggi di tutte le domande a cui è stata data risposta dividendolo poi per il numero delle domande stesse. Il punteggio massimo è 100.*
*N.B. assicurati che la linea dove viene apposto il segno dal Paziente sia effettivamente di 10 cm. In caso non lo sia, bisogna eseguire una proporzione.*
